# Smartphone-microfluidic fluorescence imaging system for studying islet physiology

**DOI:** 10.3389/fendo.2022.1039912

**Published:** 2022-11-10

**Authors:** Xiaoyu Yu, Yuan Xing, Yiyu Zhang, Pu Zhang, Yi He, Farid Ghamsari, Melur K. Ramasubramanian, Yong Wang, Huiwang Ai, Jose Oberholzer

**Affiliations:** ^1^ Department of Surgery, University of Virginia, Charlottesville, VA, United States; ^2^ Department of Molecular Physiology and Biological Physics, and Center for Membrane and Cell Physiology, University of Virginia, Charlottesville, VA, United States; ^3^ Department of Mechanical and Aerospace Engineering, University of Virginia, Charlottesville, VA, United States

**Keywords:** fluorescence microscope, Type 1 diabetes, microfluidic, smartphone, pancreatic islet, islet transplant

## Abstract

Smartphone technology has been recently applied for biomedical image acquisition and data analysis due to its high-quality imaging capability, and flexibility to customize multi-purpose apps. In this work, we developed and characterized a smartphone-microfluidic fluorescence imaging system for studying the physiology of pancreatic islets. We further evaluated the system capability by performing real-time fluorescence imaging on mouse islets labeled with either chemical fluorescence dyes or genetically encoded fluorescent protein indicators (GEFPIs). Our results showed that the system was capable of analyzing key beta-cell insulin stimulator-release coupling factors in response to various stimuli with high-resolution dynamics. Furthermore, the integration of a microfluidics allowed high-resolution detection of insulin secretion at single islet level. When compared to conventional fluorescence microscopes and macro islet perifusion apparatus, the system has the advantages of low cost, portable, and easy to operate. With all of these features, we envision that this smartphone-microfluidic fluorescence imaging system can be applied to study islet physiology and clinical applications.

## Introduction

The pancreatic islets of Langerhans are the hormone-secreting region of the pancreas and constitute 1–2% of the pancreas tissue mass. Among the four hormone-producing cells of the pancreas, beta-cells produce insulin and alpha-cells produce glucagon in response to blood glucose changes. Insulin secretion is governed by glucose metabolism, electrical activity, ion signaling, and hormone exocytosis (1), displaying complex biphasic and pulsatile kinetic profiles, and playing significant roles in the regulation of carbohydrate, fat, and protein metabolism (1).

Human islet transplant is a promising cell-based therapy for Type I diabetes (2), in which isolated islet mass and function are key factors influencing islet transplant outcomes (3, 4). Conventionally, static glucose-stimulated insulin secretion (GSIS) assay is used to determine human islet functionality. Macro islet perifusion apparatus has also been recently applied to study insulin secretion kinetics of isolated human islets due to its advantages compared to GSIS, which can measure dynamic hormone secretion. GSIS only measures” bulk” insulin secretion over stimulation time and often “ignores” dynamic nature of insulin secretion such as phase, duration, and oscillation (5). Although those macro islet perifusion systems are widely utilized, they have many limitations including expensive system setup, significant reagent consumption, high difficulty to operate, and low throughputs, as well as single parameter assay (6–8).

Compared to conventional macro techniques, microfluidic technology has been used as Islets-On-Chip (9–12). In addition to very small amounts of reagents and analyst used, the small scale allows leveraging of microscale flow phenomena, enabling the implementation of new experimental modalities that are currently not possible with available macroscale tools (12). The microfluidic transparency and planar geometry allow easy integration of bright field and fluorescence microscopy, which enables simultaneous, multiparametric, real-time imaging of islet intracellular activities and insulin secretion (7, 13–17).

Smartphone technology has been used as a highly integrated, microscopy system (18–20). It has been proven that commercially available smartphones are capable of multi-functional sensing and high-speed processing in various healthcare applications (21–24). Equipped with high resolution complementary metal-oxide-semiconductor (CMOS) cameras, smartphones can capture images at high spatial resolution. Advanced processors along with the latest wireless communication technology give smartphones the capability of executing relatively complicated image/data processing programs locally or transporting massive amounts of data to the cloud for storage and higher speed computing (25–27).

Previously, we introduced a smartphone-based brightfield digital imaging system for the quantification and comprehensive morphological characterization of isolated human islets (28). In this work, we further developed a portable, low cost, and easy-to-use smartphone and microfluidic fluorescence imaging system, which allows real-time imaging of the dynamics of intracellular beta-cell metabolic activity and ion signaling for better understanding of human islet physiology and serving as a reliable function assay before islet transplant.

## Materials and methodology

### Design of smartphone-based fluorescence imaging system

**Figure 1:**
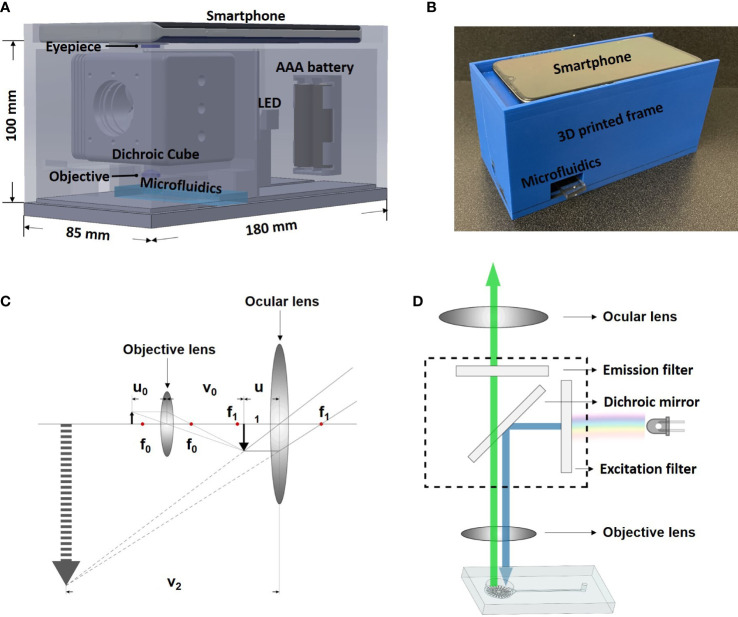
System design and working principle. **(A)** Design of the smartphone-microfluidic fluorescence microscopy system. **(B)** Assembled smartphone microscope. **(C)** Ray diagram showing the light path inside smartphone microscopes. **(D)** Optical principle of the smartphone microscope.

The smartphone microscope system consists of a smartphone (One Plus 7. OnePlus, ShenZhen, China), two external convex lenses serving as objective lens (LA1540-A, Thorlabs, Newton, NJ, USA) and ocular lens (LA1074-A, Thorlabs, Newton, NJ, USA), an illumination source (LED470L/LED505L, Thorlabs, Newton, NJ, USA), a dichroic cube (DFM2, Thorlabs, Newton, NJ, USA) containing a changeable excitation filter, an emission filter, and a dichroic mirror. These components were held together by a self-designed 3D-printed frame with dimension of 85 mm x 100 mm x 180 mm (**Figure 1A**).

As shown in **Figure 1B**, the dichroic cube holding the excitation and emission filters was placed between the smartphone camera and the microfluidic biochip. The light emitted horizontally from the illumination source passed through the excitation filter and travelled into the dichroic cube. The dichroic mirror was placed inside of the cube at an angle of 45° to reflect the excitation light vertically to be casted upon the sample. The fluorescence signal within the sample exposed to the light radiated emission light, which returned through the emission filter and was then captured by the smartphone camera (**Figure 1C**).

For optical magnification, two convex lenses (objective and ocular lens) were used. The working principle is demonstrated in **Figure 1D**. The objective lens had a focal length of 15 mm (*f*_0_). The sample was placed at 20 mm away from the objective lens (*u*_0_) so that an inverted real image (*i*_1_) was formed at the other side of the objective lens. The relationship between image distance (*v*_0_), object distance (*u*_0_), focal length (*f*), and magnification (*M*_0_) is expressed by the following linear magnification equations:


(1) $$\frac{1}{f_{0}}=\frac{1}{u_{0}}+\frac{1}{v_{0}}$$

(2) $$M_{0}=\frac{v_{0}}{u_{0}}$$

Based on the equations above, the image distance and the magnification of the objective lens were calculated to be 60 mm and 3 (*M*_0_), respectively. The focal length of the ocular lens (*f*_1_) was 20 mm. The ocular lens was placed 16 mm (*u*_1_) away from *i*_1_. Since *i*_1_ was a real image, it could be treated as a new object and a non-inverted virtual image of *i*_1_was formed at the same side of the ocular lens. Under such setup, the magnification of the ocular lens (*M*_1_) was calculated to be 5 using the following angular magnification equation:


(3) $$M_{1}=\frac{u_{1}+v_{1}}{u_{1}}$$

As such setup, the image observed by the human eye/camera was an inverted virtual image. The total magnification could be calculated by multiplying *M*_0_ by *M*_1_, resulting in 15 X magnification, as shown in the following equation.


(4) $$M_{Total}=\frac{(u_{1}+v_{1})v_{0}}{u_{1}u_{0}}$$

The total distance between the ocular lens and the objective lens was calculated as follows: *u*_1_ + *v*_0_=80 *mm*, which provided enough space to fit a dichroic cube (60 mm x 60 mm x 60 mm). Under such setup, the size of the field of view was measured to be roughly 0.8 mm x 0.8 mm.

The illumination was achieved using LEDs of corresponding excitation wavelength and were powered by 2 AAA batteries. The heat generation of the LEDs has minimal effect to the system due to its low energy consumption of ~75 mW. An Arduino Uno microcontroller (Arduino, Somerville, MA, USA) was used to receive a controlling signal from a smartphone *via* an external Bluetooth module (Shenzhen HiLetgo Technology Co., China), and switch on/off the LED with the help of a bipolar junction transistor (2N3904, Texas Instrument, Dallas, TX, US) (See **Figure S1** in **Supplement 1** for the detailed design of the LED controlling circuit).

The smartphone frame was designed using SolidWorks (SOLIDWORKS Corp, Waltham, MA, USA) and 3D printed with polylactide resin (MakerBot^®^ PLA resin, MakerBot^®^ Industries, New York, NY, USA) using a MakerBot^®^ 3D printer (MakerBot^®^ Industries, New York, NY, USA). The smartphone sat on the top of the frame while a microfluidic biochip was inserted from the side (**Figure 1A**). The dimensions of the 3D frame were 100 mm in height, 180 mm in length, and 85 mm in width.

### Design, fabrication, and validation of microfluidic biochip

**Figure 2:**
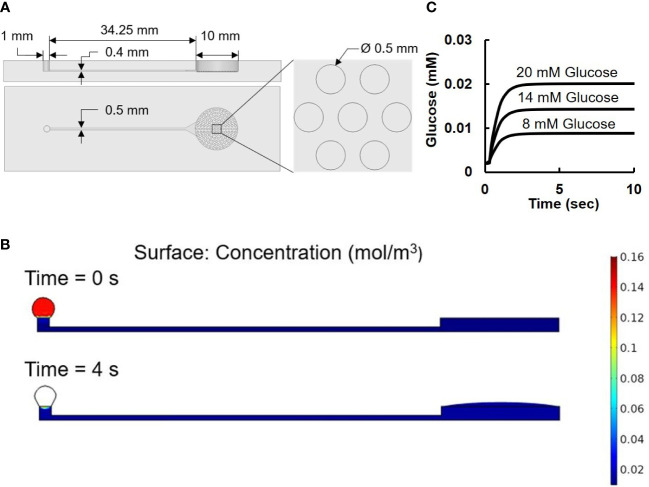
Microfluidic device. **(A)** Design of microfluidic device. The two images on the left are the front and side view of the device. The image on the right is an amplified view of the cell-loading wells. **(B)** Numerical results of surface tension driven flow in the pumpless microfluidic device: concentration level at 0 sec is set to 0.14 mol/m^3^ inside the loading droplet and 0.002 mol/m^3^ at the rest of the device. At 4 sec, the concentration level reaches its equilibrium value at 0.014 mol/m^3^. **(C)** Simulation result of glucose concentration vs. time.

Based on the passive fluid delivery method used in our previous study (7), a pumpless microfluidic biochip for islet imaging was designed and fabricated for islet perifusion and imaging. The two-layered PDMS (Polydimethylsiloxane, Fisher Scientific, Ontario, CA, USA) microfluidic device was made by soft-photolithography (See **Supplement 1** for the detailed fabrication process of microfluidic device). As shown in **Figure 2A**, the top layer contained the structures of an open-top round chamber (10 mm in diameter and 2.5 mm in depth, a total liquid volume of 196.25 µL), in which there was series of microwells (500 µm in diameter and 400 µm in depth) at the bottom for immobilizing islets. An inlet channel (400 µm in width and 40 mm in length) was designed for flow delivery, and there is an extruded structure near the islet chamber to minimize the liquid turbulence generated by flow and to prevent islets from moving.

The pumpless flow delivery was achieved through surface tension-induced pressure difference caused by the different diameters of the inlet (R_i_ of 0.5 mm) and the outlet (R_o_ of 5 mm) with R_o_/R_i_ = 10 as previously described (7).

As shown in **Figure 2A**, the droplet-based microfluidic device consisted of a drop inlet and an open-top islet immobilizing chamber connected by a capillary channel in between. With no external force and pressure driven, once a drop of glucose solution was loaded on top of the inlets, it would automatically flow towards the islets chamber compelled by the pressure generated by surface tension difference.

To further validate the microfluidic design, a COMSOL Multiphysics model was carried out using Laminar Flow and Transport of Diluted Species method (**Figure 2B**). The Navier-Stoker’s equations were utilized in the application to characterize the momentum transport of the fluid with the conservation of mass and the surface tension. A sweep function of concentrations with set parameters of 8, 14, and 20 mM were applied in the inlet droplet, while a concentration of 2 mM was defined inside of the device. The diffusion coefficient of glucose was set to be 9.59 × 10−10 m^2^/s (29). A moving triangular mesh and automatic remeshing were used to achieve accurate deformation of the droplet at the inlet. **Figure 2B** showed the simulation model at 0 second when the liquid was added to the inlet port, and at 4 second when the liquid was completely delivered to the islets.

During the loading and diffusing process, the size of the loaded drop gradually decreased as difference in surface tension drove the flow towards the islets chamber. The fluorescence measurement of islet imaging area on the microfluidic chip flushed with 10 µM Rhodamine123 showed an efficient pumpless fluid delivery of less than 3 seconds (see **Figure S2**). Computer simulation from the time-dependent study showed the glucose concentration level changes along time near the bottom center of the islet chamber (**Figure 2C**) The result showed a consistent 4 second delay among all three glucose concentrations before the chamber concentration reaches its target value, which further explains the no-contrast time zone at the beginning of the image acquisition.

### Software development for islet imaging

**Figure 3:**
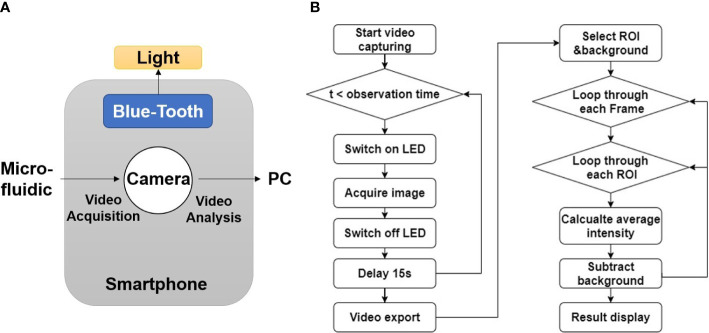
Software development for smartphone imaging system. **(A)** Diagram of software function. **(B)** Flow chart of image processing algorithm.

**Figure 3A** showed the working diagram of smartphone system. The smartphone controlled the light source *via* Bluetooth and acquire images through embedded camera. The acquired image sequences/videos were sent to PC for further processing. An overview of the software flow was shown in **Figure 3B**. The smartphone system could acquire image data at given time interval for certain amount of time. The captured image sequence/video were sent to PC for further processing. Video processing software was developed using Python 3.9.5 (Python Software Foundation, Wilmington, DE, USA) and OpenCV. The software let users to select rectangular Regions of Interest (ROIs). Since the emission band for the fluorescence biomarkers all locate around 500 nm in this work, green channel intensity was used to represent the fluorescence signal intensity. Background was subtracted to eliminate the potential interference from external light, and the average fluorescence intensity for each selected ROIs was calculated for every video frame. The results were exported to excel and plotted as fluorescence intensity vs. time in the final report for users’ review.

### Mouse islet isolation and culture

Mouse pancreatic islets were isolated and cultured as described in our previous work (17). In brief, the pancreata of 8–10-week-old C57/B6 mouse (Jackson Laboratory) were perfused with 0.375 mg/mL Collagenase P (Roche Diagnostic GmbH, Mannheim, Germany) and then digested for 12 min at 37°C. The digested pancreata were shaken vigorously for 15 s and further purified through discontinuous Ficoll gradient. The purified islets were cultured for 1-2 days before the experiment in 95% air and 5% CO_2_ at 37°C with RPMI-GlutaMAX-1640 (Gibco, Amarillo, TX, USA) supplemented with 10% fetal bovine serum (Corning, Corning, NY, USA). All procedures were approved by the Animal Care and Use Committee at the University of Virginia.

### Fluorescence imaging of islets

Fluo-4-AM (a calcium indicator. Thermo Fisher Scientific, Waltham, MA, USA) was prepared in DMSO and diluted to a final concentration of 5 µM in Krebs-Ringer (KR) buffer with 2 mM glucose (KR2). Rhodamine-123 (a mitochondrial potentials indicator. Thermo Fisher Scientific, Waltham, MA, USA) was prepared in 100% ethanol and diluted to a final concentration of 2 µM in KR2. The islets were incubated in the KR2 buffer with Fluo-4 or Rhodamine-123 for 30 min at 37°C before loading into the microfluidic device.

The genetically encoded fluorescence protein indicator (GEFPI) for calcium was delivered into islets using adeno-associated virus (AAV). To generate the virus, the sensor was first cloned into a pAAV viral transfer plasmid. The product construct was then co-transfected with pAdDeltaF6 and pAAV2/9n packing plasmids in HEK 293T cells. The crude virus was collected after four days of expression. A density gradient ultracentrifugation was performed to purify the virus. Titer of the virus was determined using qPCR-based methodology adapted from Addgene (30). Islets were handpicked into each 96-well with 100 µL complete medium and 10 µL of AAV (titer of 1x10^14^ GC/ml) were then added for a 20-hour incubation in 37°C.

A GFP filter set (452-488 nm/506-545 nm, Iridian spectral technology, Ottawa, ON, Canada) was used to observe Fluo-4 fluorescence dye and the GEFPI. The smartphone camera was set to 200 ISO and 1/10 shutter time, and images were captured at 15 s intervals. The chamber was filled with KR2 buffer initially, and the fluorescence-labeled islets were then carefully loaded into the open top chamber in the microfluidic device using a 20 µL pipette. KR buffer containing varying glucose concentrations (5, 14, or 20 mM) and KCL (30 mM) were introduced to islets through the inlet. Tolbutamide (Sigma-Aldrich, St. Louis, MO, USA) and Diazoxide (Sigma-Aldrich, St. Louis, MO, USA) were prepared in KR2 buffer with a final concentration of 250 µM and 200 µM, respectively.

A YFP filter set (Iridian spectral technology, Ottawa, ON, Canada) was used to observe the Rhodamine-123. The filter set had an excitation band of 489-505 nm, and an emission band of 524-546 nm. The camera settings used for Rhodamine-123 was identical to the ones used for Fluo-4 and the GEFPI.

### Single islet insulin secretion

A single islet was loaded into the microfluidic device and incubated in KR2 buffer for 30 min and at which time the supernatant was collected to serve as the basal level. After an additional 30 min incubation under 14 mM glucose, the supernatant was collected again as the stimulated sample. Insulin concentrations were determined using Mouse Insulin ELISA kit (Mercodia, Winston Salem, NC, USA) using a plate reader (Biotek Synergy H1, BioTek U.S., Winooski, VT, USA).

### Statistical analysis

Data were expressed as mean ± SD. For fluorescence intensity measurement and GSIS, all experiments were conducted under each stimulation protocol (n = 5, biological replicates, * p<0.05, ** p<0.01, *** p<0.001). Data were analyzed using Python 3.9.5, and plots were generated using Prism 9 (GraphPad, San Diego, California, USA).

## Result and discussion

### Imaging resolution validation of optical system

**Figure 4:**
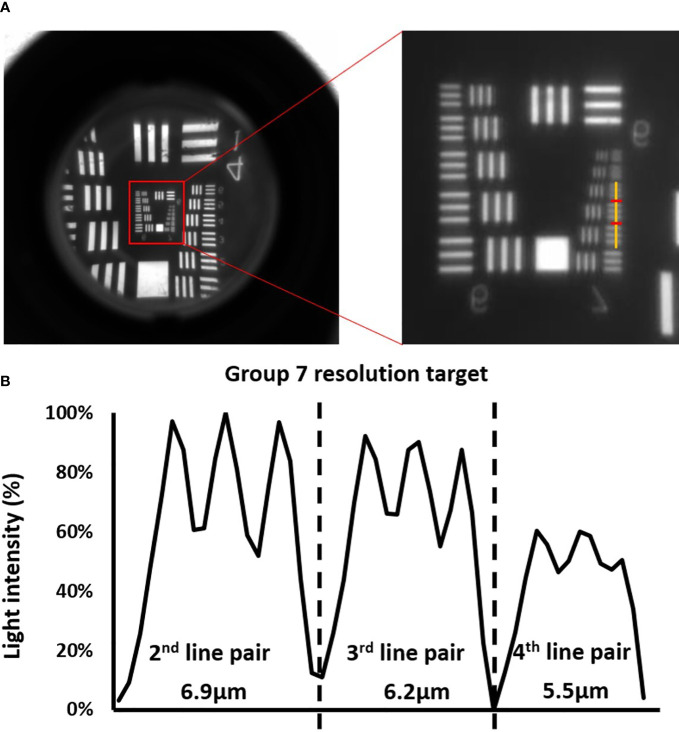
Validation of the optical system. **(A)** Images of the resolution targets. The lowest resolved groups (Group 7) are highlighted using red box in the image on the left, and the image on the right is the zoom -in image of the highlighted area with a yellow vertical line cut in the middle of the horizontal line pairs. **(B)** The corresponding cross-sectional plot of the intensity along the yellow vertical line.

Spatial resolution of the imaging system was measured using a resolution target purchased from Edmund Optics (Edmund Optics, Barrington, NJ, USA). **Figure 4** presented validation result for the imaging system mentioned in chapter 2.1. All the lines up until the 3^rd^ line pair (6.2 µm) in group 7 could be clearly distinguished. Starting from the 4^th^ line pair (5.5 µm) in group 7, the contrast between the bright and dark line could no longer be well-identified. **Figure 4B** plotted the light intensity along the yellow line. The curve quantified the level of contrast of the 2^nd^ – 4^th^ line pairs in group 7, and the average difference between the peak values in bright lines and the bottom values in dark lines descended from ~20% to ~10%. Both **Figures 4A, B** showed that the 3^rd^ line pair in group 7 was a good representation of the maximum resolvable resolution, which was 6.2 µm. In **Figure S3**, the resolution validation result of imaging system with not magnification was presented. The result indicated that the maximum resolvable resolution was ~99 µm, meaning that the magnification setup mentioned in chapter 2.1 brought a ~15 times enhancement on spatial resolution. Given that islets had diameters larger 50 µm in most cases, such enhancement ensured that the imaging system has enough resolution for islets imaging.

### The effects of photo-bleach and shear stress on islet calcium signaling

**Figure 5:**
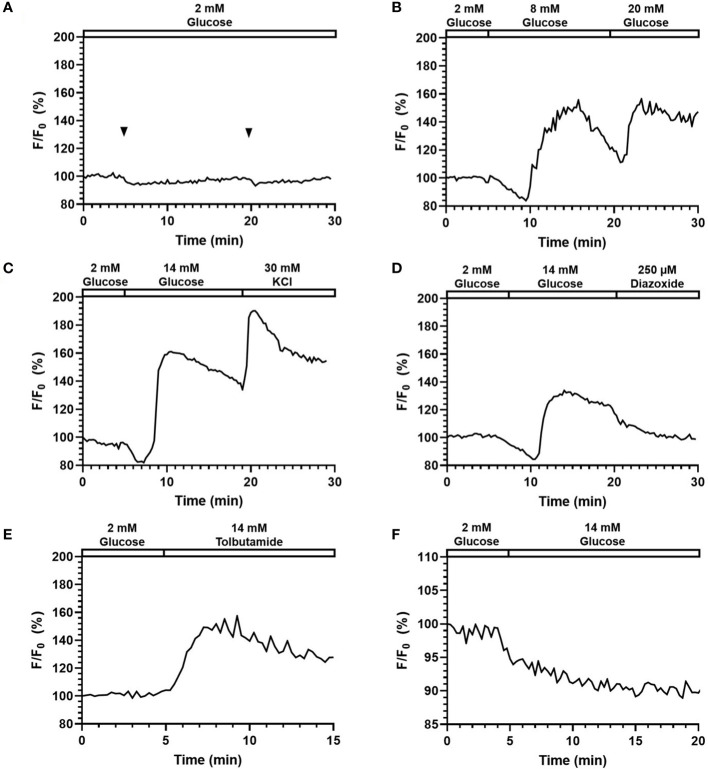
Perifusion and imaging of Fluo-4 ([Ca^2+^]_i_) and Rhodamine123 (Ψ_mito_) labeled mouse islets. Representative tracings of Fluo-4 [Ca^2+^]_i_ in mouse islets following **(A)** continuous addition of 2 mM glucose, **(B)** glucose stimulation at varying concentration (8 and 20 mM), **(C)** 14 mM glucose and 30 mM KCl stimulation, **(D)** 14 mM glucose and 200 µM Diazoxide stimulation, **(E)** 250 µM Tolbutamide stimulation. N = 5. **(F)** Representative tracings of Ψ_mito_ in mouse islets following 14 mM glucose stimulation. N = 5.

The photo-bleach in fluorescence imaging caused by the overwhelming exposure can lead to loss of fluorescence signals and affect the analysis results. Also, the shear stress generated from fluid flow in a microfluidic channel may induce calcium ion influx in pancreatic beta-cells (31). Therefore, we monitored the intracellular fluorescence calcium signals in response to the continuous addition of flow solution over 30 min. As shown in **Figure 5A**, the intensity curve was almost flat, indicating that the photo-bleach effects were neglectable. Minor fluctuations could be observed at 5 min and 20 min when flow was delivered, but the fluctuations were very minimal (< 3%), meaning that the shear stress from the pumpless flow delivery did not have significant impact on the fluorescence signal acquisition.

### Intracellular insulin stimulator-secretion coupling factor in response to stimuli

Chemical dyes, fluorescence, and confocal imaging are often employed to study beta-cell intracellular activities such as calcium influx, mitochondrial potential changes, zinc release kinetics, ROS production, and many others (32–35). In this study, we used Fluo-4 and Rhodamin-123 to monitor cellular calcium influx and mitochondrial potentials, respectively.

**Figures 5B-E** shows the representative responses of Fluo-4-labelled islets to various stimuli and inhibitors. In **Figure 5B**, the fluorescence signal was increased to 158.2% (164.7% ± 10.8) in response to 8 mM glucose, and 165.0% (163.1% ± 15.8) in response to 20 mM glucose. Importantly, the system can clearly detect a typical biphasic calcium pattern under glucose stimulation. When comparing the two responses, the two peaks had similar height, yet while the second one (20 mM) managed to maintain a high level, the first response (8 mM) started to decrease within ~2min and reached to 119.2% (114.5% ± 12.0) before the second stimulus was added. These results suggested that delivery of stimuli to islets can be achieved with the proper concentrations through the surface tension driven liquid delivery process. Under 14 mM glucose and 30 mM KCl stimulation (**Figure 5C**), fluorescence intensity first experienced a slight drop to 85.2% (86.8% ± 2.9) corresponding to phase 0, and then started to quickly increase until reaching the highest value of 167.6% (157.0% ± 9.5). The fluorescence intensity maintained a high level, and further increased to 197.81% (183.5% ± 17.0) after the 30 mM KCl stimulation.

As shown in **Figure 5D**, the fluorescence intensity increased to 151.8% (141.2% ± 17.0) in response to 250 μM Tolbutamide (a K_ATP_-channel closer). In **Figure 5E**, when 200 μM Diazoxide (a K_ATP_-channel opener) was added after 14 mM glucose, the fluorescence intensity dropped to 108.8% (114.4% ± 13.2) from 133.8% (145.3% ± 14.7) at 2 min and continued dropping at a relatively slower rate afterward.

To test the system’s adaptability on a different fluorescence spectrum, Rhodamine-123 (*Excitation* 507 nm, *Emission* 529 nm) was also used to measure beta-cell mitochondrial potentials changes. As shown in **Figure 5F**, fluorescence intensity dropped to 89.2% (88.5% ± 2.8) when stimulated with 14 mM glucose at 5 min. The result captured by our system correctly reflected the mitochondrial potential hyperpolarization post glucose stimulation, which suggested that the smartphone system was able to monitor fluorescence signals of different wavelengths.

### Fluorescence imaging of islets labeled with GEPFIs

One of the challenges in islet biology is to visualize biomolecules in their natural environment in real-time and in a non-invasive fashion, so as to gain insight into their physiological behaviors and highlight alterations in pathological settings. GEFPIs constitute a class of imaging agents that enable visualization of biological processes and events directly *in situ*, preserving the native biological context and providing detailed insight into their localization and dynamics in cells.

**Figure 6:**
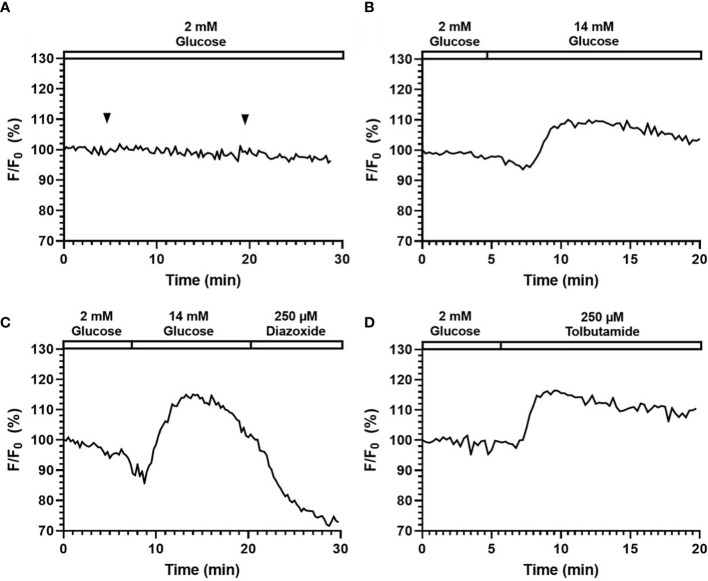
Perifusion and imaging of GEPB labeled mouse islets. Representative tracings of [Ca^2+^]_i_ in mouse islets following continuous addition of **(A)** 2 mM glucose, **(B)** 14 mM glucose stimulation, **(C)** 14 mM glucose and 200 µM Diazoxide stimulation, **(D)** 250 µM Tolbutamide stimulation. N = 5.

Here, a calcium GEFPI was expressed in islet cells and then imaged using the smartphone system. Similar to Fluo-4, we did not observe significant photo-bleach or calcium fluctuation related to light exposure and fluid delivery (**Figure 6A**). As shown in **Figure 6B**, the fluorescence intensity of the fluorescence signal of calcium GEFPI in response to 14 mM glucose increased to 110.0% (113.1% ± 4.8) after a minor drop, which was relatively smaller when compared to that of Fluo-4. This was followed by a sharp increase after glucose stimulation was presented indicating that the smartphone system was able to capture the signal emitted by the biosensor and identify the difference between pre- and post- glucose stimulation. When stimulated by Tolbutamide, the fluorescence intensity increased to 116.4% (115.1% ± 2.1) (**Figure 6C**). [Ca^2+^]_i_ influx induced by 14 mM glucose can be reduced by 200 µm Diazoxide from 116.9% (115.9% ± 2.3) to 83.1% (87.9% ± 7.6) (**Figure 6D**).

### Single islet insulin secretion

**Figure 7:**
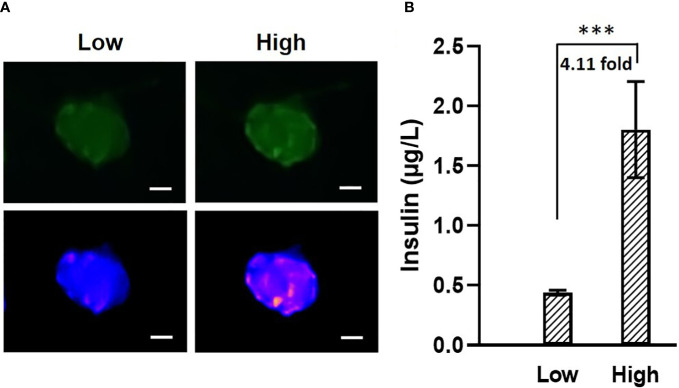
**(A)** Images of fluorescence labeled mouse islets in response to low (2 mM, left) and high (14 mM, right) glucose. The two images on top are the original images, The two at the bottom are transformed into “blue-purple-yellow” color map for better visualization. Scale bar = 50 µm. **(B)** Mouse islets insulin secretion in response to 2 mM and 14 mM glucose. N = 5, *** p<0.001.

It has been known that islets and stem-cell derived islet biologics have widely varying insulin secretion capacity. Traditional static glucose stimulated insulin secretion (GSIS) assay often measures insulin output from pooled islets, which is imitated to measuring single islet insulin secretion. In conjunction with fluorescence imaging capability, we performed single islet insulin secretion in response to glucose challenge. Images were taken before and after glucose stimulation (**Figure 7A**), where obvious changes in fluorescence intensity could be observed. Meanwhile, the ELISA result (**Figure 7B**) showed that our microfluidic system can actually measure single islet insulin secretion capability in response to 14 mM glucose stimulation with insulin secretion index of 4.11 ± 0.73. As such, the microfluidic single islet assay has potential advantages against the pooled GSIS assay by allowing a number of technical replicates while maintaining or reducing the overall number of islets required for GSIS. Furthermore, individual islet basis measurements will allow for identification of a sub-par performing islet population within an entire preparation and for quantification of the standard deviation of insulin secretion response of individual islets in a preparation.

## Conclusion

In this study, we introduced a novel smartphone-microfluidic fluorescence imaging system to study the physiology of islet beta-cells. By trapping islets in a customized surface tension driven pumpless microfluidic device, we managed to use the smartphone camera to visualize mouse islets labeled with various fluorescence indicators and detected physiological insulin stimulator-secretion coupling factors, showing decent fluorescence signaling and signal vs. noise ratio in response to different stimuli/inhibitors. Our system can also achieve adequate resolution of single islet insulin secretion.

Although there are many smartphone-based fluorescence imaging system, to the best of our knowledge this is the first smartphone-based fluorescence imaging system specifically designed for islet function analysis. When compared to conventional fluorescence-microscopic microfluidic systems, the portable system allows an easier setup and a much lower cost (See **Table S1** in **Supplement 1** for the estimated total cost of the system). Importantly, it can achieve comparable resolution and detection of fluorescence signals when compared to standard fluorescence microscope. One limitation with the current system setup is that current setup can only work with one fluorescence spectrum at a time. However, this problem can be resolved in the future by implementing filter switch functionality into the system with the help of a motorized filter wheel.

In conclusion, the presented smartphone-microfluidic imaging system reveals a possible implementation of portable, low-cost fluorescence microscope to study the insulin secretion kinetics of islet beta-cells and insulin stimulator-secretion coupling factors. In future, we will incorporate the concepts of big data and machine learning to transform the system into a front-end device for islet data gathering, while embedding a fully trained analysis model such that the system can generate assistive results on human islet functionality. Eventually, it can serve as an assay that has predictive value for islet graft function for Type I diabetes and replace *in vivo* animal model currently used to predict long-term islet transplant outcomes (36).

## Data availability statement

The raw data supporting the conclusions of this article will be made available by the authors, without undue reservation.

## Author contributions

YW, XY, and YX designed the experiments. XY developed the software, designed the system frame, and performed most of the experiments. XY and YX designed the microfluidic device and configured the optical setups. PZ designed and performed the simulation for microfluidic device. YH performed cell preparation for the experiments. YZ performed the islet transduction process. XY and YX analyzed the data. All authors contributed to discussions and manuscript preparation. XY, YX, and YW wrote the manuscript with input from all authors. FG assisted with manuscript revision. MR, HA, YW, and JO supervised the project. All authors contributed to the article and approved the submitted version.

## Funding

NIH R01 DK122253 (HA, JB, YW); NIH NIDDK R25 DK105924-01 (JB); UVA Launchpad for diabetes (HA, JB, YW).

## Acknowledgments

The authors would like to acknowledge the help from MAE Rapid Prototyping Lab and Machine Labs in fabricating the 3D-printed frame of the system.

## Conflict of interest

The authors declare that the research was conducted in the absence of any commercial or financial relationships that could be construed as a potential conflict of interest.

## Publisher’s note

All claims expressed in this article are solely those of the authors and do not necessarily represent those of their affiliated organizations, or those of the publisher, the editors and the reviewers. Any product that may be evaluated in this article, or claim that may be made by its manufacturer, is not guaranteed or endorsed by the publisher.
