# Supplementary material for: Smartphone-microfluidic fluorescence imaging system for studying islet physiology
Source: Front Endocrinol (Lausanne). 2022 Nov 10;13:1039912. doi: 10.3389/fendo.2022.1039912 (PMC9684609; doi:10.3389/fendo.2022.1039912)
Supplement: Supplementary file 1 [file DataSheet_1.docx]

Supplementary Material

# Device fabrication

The microfluidic device was fabricated using soft- photolithography protocol. The device was designed in AutoCAD and printed on a transparent film (Fineline Imaging, CO) with high resolution (16000dpi). SU-8 2150 photoresist (Microchem, MA, USA) was pre-spun to 400 µm for both top and bottom layers on two silicon wafers. The printed film functioned as a photomask, was placed on top of the photoresist, and was exposed to UV light such that the selected region of the spun photoresist could cross-link. Finally, uncross-linked photoresist was washed away by cleaning the wafer with SU-8 developer solution. The parts left on the wafer should resemble the features printed on the film. Once the SU-8 negative mold master was fabricated, PDMS was poured on top to generate positive feature at desired thickness. The PDMS was then placed at 80°C for 6 hrs for solidification, and the parts with features were cut and peeled off from the mold. Punchers of certain sizes were used to puncture inlets and chambers on the top PDMS layer. The two layers were bonded together by treating with oxygen plasma followed by 2hr of heating at 80 °C while pressed together with weight.

Supplementary material is not typeset so please ensure that all information is clearly presented, the appropriate caption is included in the file and not in the manuscript, and that the style conforms to the rest of the article. To avoid discrepancies between the published article and the supplementary material, please do not add the title, author list, affiliations or correspondence in the supplementary files.

# LED control circuit design

The circuit was designed and transferred to PCB layout using Multisim. The PCB layout was then sent to OSH Park (Portland, OR, USA) for fabrication. The circuit design is shown in the following figure. The design utilized a saturated bipolar junction transistor (BJT, 2N3904) to switch the LED, the collector of which was connected to the LED followed by a pull-up resistor. A voltage regulating circuit was connected to the other side of the pull-up resistor. This part of the circuit utilized a linear regulator (LM1117), which could generate a constant 1.25V between the OUT node and the GND node. This voltage was amplified by connecting the GND node to the middle of a voltage divider, and the amplification ratio could be adjusted by tuning the resistor values of the voltage divider. The capacitors used in the circuit were to eliminate the potential influence caused by external noises.

**
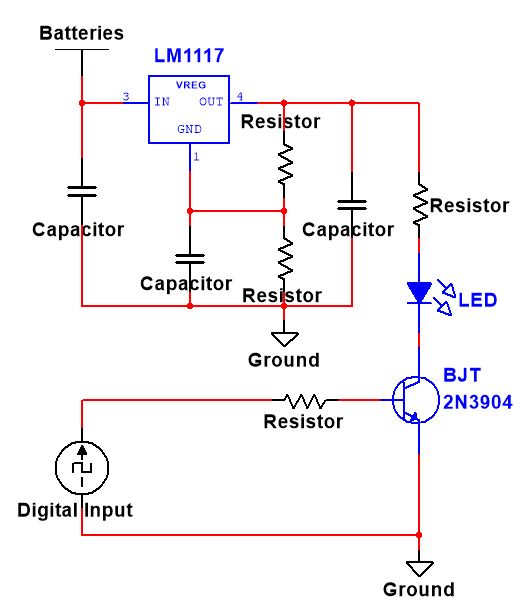
**

**Supplementary Figure 1.** Circuit design for LED control and power supply.

# Estimated cost of the system

The table below includes the estimated cost of the system.

| 3D printed frame | | $200 |
| --- | --- | --- |
| Microfluidic device | | $10 |
| Optical components | Dichroic cube | $500 |
|  | Fluorescence filter set | $400 |
|  | Objective lens | $30 |
|  | Ocular lens | $30 |
| Electrical components | LEDs | $60 |
|  | Batteries | $2 |
|  | Arduino Uno | $20 |
|  | Bluetooth module | $10 |
|  | Voltage regulator | $2 |
|  | PCB board | $20 |
|  | Resistors + capacitors | $1 |
|  | BJT | $5 |
| Total | | $1,290 |

# Pumpless fluid delivery

The islet chamber of microfluidic biochip filled with 100 µL of KR2 buffer was flushed by 10 µL KRB containing 10 µM Rhodamine 123 at 0 sec. The smartphone system with YFP filter was used to monitor the fluorescence intensity of the viewing area. The normalized fluorescence intensity vs. time was plotted in the following figure. F/F_0_ reached its peak at ~3.8 sec.


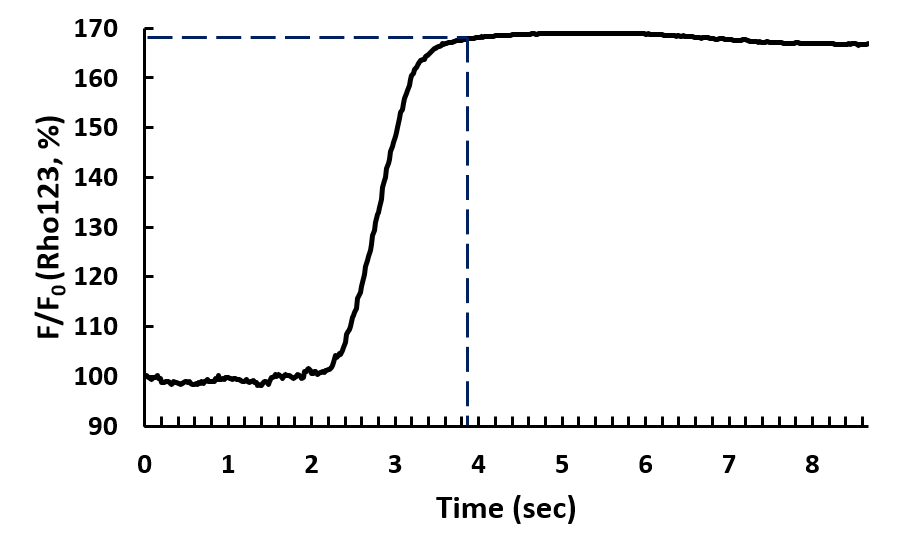


**Supplementary Figure 2.** Pumpless fluid delivery monitored using fluorescence.

# Imaging of resolution target

The following figure presented resolution target imaged under M = 1 setup. As compared to the magnified image in Fig.4A, the magnification ratio was calculated to be ~16.7, which roughly matched the calculated magnification ratio.


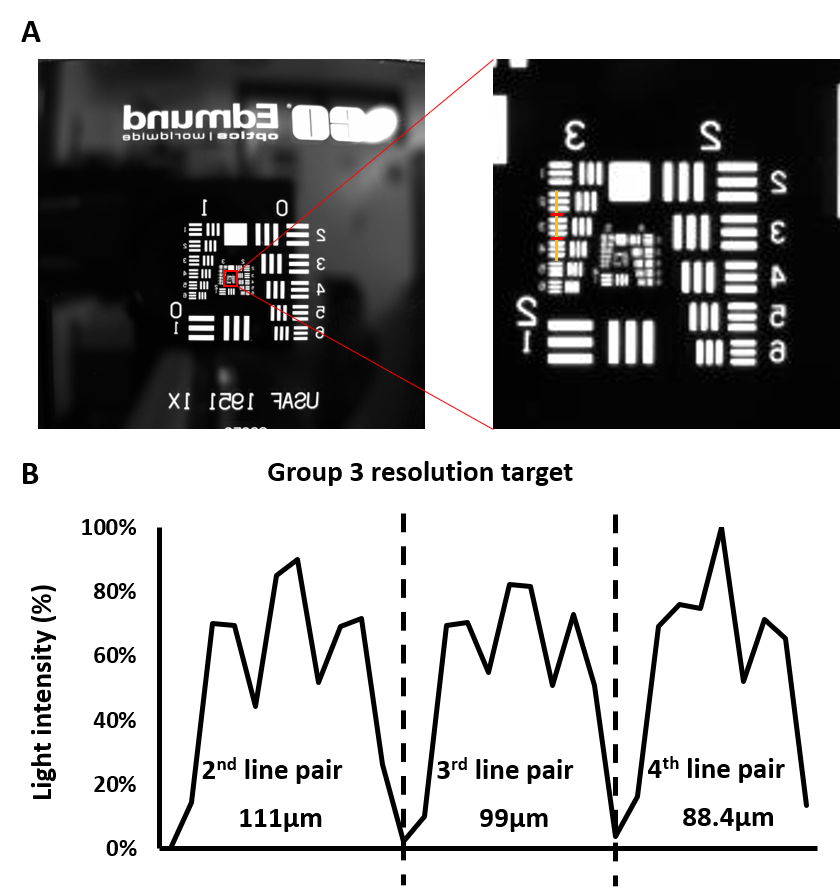


**Supplementary Figure 3 A)** Images of the resolution targets when M = 1. The lowest resolved groups (Group 3) are highlighted using red box in the image on the left**,** and the image on the right is the zoom -in image of the highlighted area with a yellow vertical line cut in the middle of the horizontal line pairs. **(B)** The corresponding cross-sectional plot of the intensity along the yellow vertical line in (B).
